# Early Defervescence and SARS Recovery

**DOI:** 10.3201/eid1003.030500

**Published:** 2004-03

**Authors:** Jann-Tay Wang, Jiun-Ling Wang, Chi-Tai Fang, Shan-Chwen Chang

**Affiliations:** * National Taiwan University Hospital, Taipei, Taiwan

**Keywords:** SARS, atypical presentation, temporary defervescence

**To the Editor:** Severe acute respiratory syndrome (SARS) is an emerging disease first recognized November 2002 (*1*). Previous studies show patients with probable SARS on ribavirin and steroid therapy may experience a biphasic course, with clinical symptoms and changes shown on chest x-rays increasing in the second week of disease (*2*). We report a patient with probable SARS who had temporary defervescence for 7 days before rapidly progressing to respiratory failure.

The patient was a 54-year-old female nursing aide for a patient with fever and pneumonia who was diagnosed with probable SARS on the basis of the criteria proposed by World Health Organization (WHO) (*3*). Our patient did not have underlying disease, but fever of 38.6°C developed on May 10, 2003, a total of 3 days after her last contact with the patient she was caring for. Mild myalgia was noted. She was admitted that day with suspected SARS. Initial chest x-ray results were normal. Hemogram showed a normal leukocyte count with mild lymphopenia (absolute lymphocyte count 0.84 x 10^9^/L) and a normal platelet count (253 x 10^9^/L). Initial serum aspartate aminotransferase (AST) and alanine aminotransferase (ALT) levels were 38 U/L and 20 U/L, respectively (normal <35 U/L). Serum creatinine kinase (CK) level was 84 U/L (normal <190 U/L). Serum C-reactive protein (CRP) level was elevated (3.49 mg/dL; normal <0.8 mg/dL). Serum sodium level was normal.

After admission, she received oral ribavirin, 1,000 mg/day. No other antimicrobial agent was administered. Her fever persisted for 2 days, and she became afebrile spontaneously on May 13, 2003. The result of reverse transcription–polymerase chain reaction (RT-PCR) for SARS-associated coronavirus (SARS-CoV) on a throat swab specimen performed on May 10 was negative. All other testing, including blood culture; virus isolation; and serologic tests for SARS-CoV, chlamydia, mycoplasmas, rickettsia, influenza virus, parainfluenza virus, adenovirus, respiratory syncytial virus (RSV), and coxsackie virus were also negative. She did not take any nonsteroid antiinflammatory drugs (NSAIDs) or steroids during this period. Her chest x-ray results on May 13 remained normal. Other laboratory testing on May 13 showed resolution of lymphopenia, a lower serum CRP level (2.48 mg/dL), but an elevated lactate dehydrogenase (LDH) level (627 U/L; normal <460 U/L).

During the next 4 days, she remained afebrile. Results of a repeated chest x-ray on May 16 were still normal. The serum CRP level decreased to 2.29 mg/dL. However, borderline leukopenia (4.45 x 10^9^/L), borderline thrombocytopenia (167 x 10^9^/L), an elevated serum CK level (238 U/L), hyponatremia (128.2 mmol/L), and a progressively elevated serum LDH level (1,138 U/L) were noted. Because she had been afebrile for 5 days, she was discharged on May 17. After discharge, she continued to take ribavirin and be quarantined at home. Unfortunately, fever and rapidly progressive dyspnea developed on May 20. On the same day, chest x-ray showed diffusely increased infiltration over all lung zones. Hemogram showed leukocytosis (leukocyte count 14.03 x 10^9^/L). Serum CRP level was elevated to 12.7 mg/dL. Serum sodium level was 129.5 mmol/L. Serum AST level was 135 U/L, serum CK level was 71 U/L, and serum LDH level was 1,719 U/L. All blood cultures and sputum culture for bacteria yielded nothing.

She was intubated on May 21 for respiratory failure. Under the assumption of probable SARS, she was given high-dose methylprednisolone (120 mg/day), and her clinical condition stabilized soon after. The results of RT-PCR for SARS-CoV on a throat swab specimen performed on May 21 were positive. The results of immunofluorescent assays testing for immunoglobulin (Ig) M and IgG against SARS-CoV (performed in the research laboratory at National Taiwan University Hospital on May 21 and 27) were all positive (both IgM titers >1:10; both IgG titers >1:1,000). Sputum culture and Gram stain were both negative. Urine tests were also negative for pneumococcal and *Legionella* antigens. Other serologic tests, including those for chlamydia, mycoplasmas, rickettsia, influenza virus, parainfluenza virus, adenovirus, RSV, and coxsackie virus were still negative. The ventilator was removed on June 9.

A previous report pointed out the great variety in the clinical course of SARS (*4*). We emphasize that even a patient with suspected SARS who became afebrile in the first week and remained so for 7 days without steroid or NSAID treatment still risks deterioration in the second week, as long as some laboratory values remain abnormal. Therefore, defervescence, even up to 7 days, may not be the single indicator for discharging SARS patients. Obtaining normal results for previously abnormal laboratory parameters, including hemogram, CRP, CK, AST, ALT, and LDH levels should be considered when deciding whether a patient can be safely discharged (*5*).
